# Consideration of future consequences (CFC) serves as a buffer against aggression related to psychopathy

**DOI:** 10.1371/journal.pone.0203663

**Published:** 2018-09-12

**Authors:** Yongping Zhao, Jia Wei, Yuanshu Chen, Lingxiang Xia

**Affiliations:** 1 Faculty of Psychology, Southwest University, Chongqing, China; 2 School of Educational Science, Sichuan Normal University, Chengdu, Sichuan, China; 3 School of Life Science and Technology, University Electronic Science and Technology, Chengdu, Sichuan, China; Technion Israel Institute of Technology, ISRAEL

## Abstract

Psychopathy is the most notorious trait in the Dark Triad, and it is strongly linked to many kinds of aggressive behaviors. However, not every individual who is characterized by psychopathy engages in aggression, which suggests that certain factors may attenuate the intensity of the relations between psychopathy and aggression. The purpose of the current study was to explore the protective roles of the consideration of future consequences (CFC) (high CFC-Future and low CFC- Immediate) in attenuating aggression related to psychopathy using proactive aggression (Study 1) and cyber-aggression (Study 2) behavior indexes. College students (Study 1; *N* = 1,058) and adults (Study 2; *N* = 350) voluntarily participated in this study. The results demonstrated that the relationship between psychopathy and aggressive behaviors was moderated by CFC-Future and CFC-Immediate. Individuals with high psychopathy scores who also had high CFC-Future scores or low CFC-Immediate scores exhibited less proactive aggression (Study 1) and left fewer aggressive online comments on news websites (Study 2). The results of the present study suggested that CFC serves as a buffer against aggression related to psychopathy and may extend the knowledge of the relationship between psychopathy and aggression.

## Introduction

Psychopathy is the most notorious personality trait of the Dark Triad [1] and is characterized by a lack of empathy, low guilt, callousness, impulsive thrill-seeking, and irresponsibility [2]. Psychopathy is correlated with dysfunctional impulsivity [3] and limited self-control [4]. Abundant studies have shown that psychopathy predicts many kinds of aggressive behaviors [5–7], such as physical aggression [8–10], relational aggression [11, 12], verbal aggression [8], proactive aggression [6, 13, 14] and cyber-aggression/bullying [15–18].

Proactive aggression, as a traditional aggressive subtype, is an unprovoked, purposeful and goal-directed aggressive behavior that is motivated by the perpetrator’s expectation of a positive outcome [19]; this type of aggression may be further categorized as physical, relational or verbal aggression [20]. Cyber- aggression/bullying, as a modern form of aggression, is defined as intentionally harmful behavior performed by the use of electronic communication to an individual or a group of individuals who perceive(s) these acts as offensive, derogatory, harmful or unwanted [21]. Both proactive aggression [6, 13] and cyber-aggression/bullying [16–18] were more strongly predicted by psychopathy than by narcissism or Machiavellianism in the Dark Triad. Thus, the present study used proactive aggression and cyber-aggression as the indexes of aggression to assess the relationship between psychopathy and aggression.

In contrast to psychopathy, the consideration of future consequences (CFC) [22] seems to be one kind of protective personality trait related to decreases in aggression because CFC is linked to a future time perspective [23, 24], which may directly lead to future-oriented individuals being more capable of controlling aggressive reactions [25]. CFC represents stable individual differences regarding the degree to which individuals focus more on the future vs. the immediate consequences of a potential behavior [22]. Individuals who show a low CFC are expected to emphasize their immediate needs and concerns and may act to satisfy these immediate needs. In contrast, individuals who show a high CFC are expected to focus on the potential outcomes of their behaviors and allow distant goals to guide their present actions. CFC is regarded as a unidimensional conceptualization of temporal orientation that reflects future and immediate consequences of present actions on the original scale [22]. Immediate items have reverse scores on the 5-point scale.

However, the unidimensional conceptualization of CFC incorrectly assumes that individuals who strongly disagree with one immediate-focused item would strongly agree with the converse of the item, or vice versa [26]. Many studies have confirmed the bi-dimensional constructs of CFC [26–29]. Thus, considering immediate consequences (CFC-I) and considering future consequences (CFC-F) are two different dimensions. High CFC-I individuals are more likely to seek smaller, immediate rewards [29], while high CFC-F individuals are more likely to seek larger and less certain immediate rewards [27]. Accordingly, CFC-F has been suggested to reflect a future orientation, while CFC-I has been suggested to reflect a present orientation [29]. Many empirical studies have shown that future orientation negatively predicted aggression and bullying [30–35] as well as violent behaviors [36, 37]. Present orientation positively predicted aggression [38, 39]. Based on previous analyses, we may infer that CFC-I positively predicts aggression, and CFC-F negatively predicts aggression. However, there remains a shortage of direct research on the role of CFC as a bi-dimensional construct in decreasing aggression. Thus, the present study aimed to explore the protective role of CFC as a bi-dimensional construct against aggression.

Although psychopathy seems to be closely related to aggression, it does not indicate that all individuals with high psychopathy will always exhibit high aggression. Human beings are complex creatures, and individuals commonly have different or contradictory traits [40]. These traits may interact to decrease aggressive behavior. CFC [22] may be one kind of protective personality trait for decreasing aggression triggered by psychopathy. This inference was based on several indirect pieces of evidence. First, previous studies have shown that the relationship between certain personality variables and behaviors could be moderated by other individual variables. For example, self-reported resilience attenuated the relation between callousness (an important component of psychopathy) and aggression [41]. Studies have also indicated that the links between psychopathy and aggression are moderated by several individual variables, such as intelligence [7] and mentalization [42]. Specifically, more intelligent psychopaths may be less inclined to use aggression because they can use their cognitive resources to devise nonviolent means to obtain what they want. High levels of mentalization may decrease proactive aggression in individuals with psychopathic traits. Second, the predictive effects of impulsive sensation seeking and self-control (the other two key elements of psychopathy) on delinquency [43] and risky behaviors [44], respectively, are moderated by CFC. These results suggested that CFC (high CFC-F and low CFC-I) may buffer the effect of psychopathy on aggressive behaviors.

Based on the above mentioned analyses, this study examined the independent and interaction effects of psychopathy and the consideration of future consequences (CFC-I vs. CFC-F) on proactive aggression using data from college students (Study 1) and on cyber-aggression using data from adults (Study 2) to (a) explore the protective role of CFC against aggression and (b) explore the moderating effects of CFC in attenuating aggression related to psychopathy.

## Study 1

In Study 1, we aimed to test the following hypotheses: 1) psychopathy and CFC-I positively predict proactive aggression, and CFC-F negatively predicts proactive aggression; and 2) CFC-I positively moderates the relationship, and CFC-F negatively moderates the relationship between psychopathy and proactive aggression.

### Method

#### Participants and procedure

In total, 1058 college students (612 females) from six different universities participated in the study. The participant ages ranged from 17 to 26 years (M = 20.42, *SD* = 1.09). The participants were instructed to complete paper-pencil surveys, including the psychopathy subscale of the Dirty Dozen scale [45], the CFC scale [22], and the proactive aggression subscale of the reactive-proactive aggression questionnaire [46]. According to the suggestions of previous research [47], we used procedural remedies to control likely common method biases. The first two scales were presented in two orders, and the participants responded anonymously to the scales. Finally, the participants provided their demographic data, including age and sex. Verbal informed consent was obtained from all students prior to the survey, and this research was approved by the Ethics Committee at the Faculty of Psychology, Southwest University, China.

#### Materials

The psychopathy subscale of the Dirty Dozen scale [45] was used. The subscale included four items (e.g., “I tend to lack remorse”) and was measured with a response scale from 1 (strongly disagree) to 7 (strongly agree). The Cronbach’s α value for the present sample was 0.67.

The CFC scale [22] was used. This scale included two constructs: CFC-I and CFC-F [28, 29]. The CFC-I included seven items (e.g., “I only act to satisfy immediate concerns, figuring the future will take care of itself”), and the CFC-F included five items (e.g., “I consider how things might be in the future and try to influence those things with my day to day behavior”). The participants rated each statement based on their own behavior on a scale of 1 (extremely uncharacteristic) to 5 (extremely characteristic). The Cronbach’s α of the two subscales for the present sample were 0.74 for CFC-I and 0.65 for CFC-F.

The proactive aggression subscales of the reactive-proactive aggression questionnaire [46] were used. This scale included 12 items related to physical and verbal aggression (e.g., “how often have you hurt others to win a game?”). Each item was rated as 0 (never), 1 (sometimes), or 2 (often) for the frequency of occurrence. The Cronbach’s α for the present sample was 0.88.

#### Statistical analysis

Statistical analyses were performed using SPSS Version 22. We conducted Harman’s single factor test [47] to examine whether the results were affected by common method bias. All the scale items were subjected to exploratory factor analysis to determine whether a single factor emerged. The results indicated six factors with eigenvalues greater than 1.0 and the first factor accounted for 18.79% of the accumulation contribution rate (< 40%). Our results indicate that common method biases were not a problem in Study 1. Descriptive analyses, including correlation coefficients, the means, and standard deviations, were calculated to assess the overall patterns of psychopathy, CFC-I, CFC-F, and proactive aggression. To test the main and moderating effects, gender, psychopathy, CFC-F and CFC-I were included to conduct hierarchical regression analysis. Independent sample t-tests were used to assess gender differences.

#### Results and discussion

The descriptive statistics of the sample are presented in Table 1.

**Table 1:** Zero-order correlations, means, and standard deviations.

	**1**	**2**	**3**	**4**	**5**	**6**
**1 DT-P**	1					
**2 CFC-I**	-0.11**	1				
**3 CFC-F**	-0.16**	0.013	1			
**4 AGGRE**	0.33**	0.026	-0.25**	1		
**5 Gender**	0.20**	0.028	-0.096**	0.18**	1	
**6 Age**	-0.017	0.013	0.026	-0.009	0.11**	1
***M***	2.17	2.82	3.53	0.096	0.42	20.42
***SD***	1.00	0.59	0.59	0.22	0.49	1.09

DT-P, psychopathy; CFC-F, CFC-Future; CFC-I, CFC-Immediate; AGGRE, proactive aggression;

**p < 0.01.

Table 1 showed that gender and psychopathy positively but CFC-F negatively and significantly was related to proactive aggression. To test the main and moderating effects, gender, psychopathy, CFC-F and CFC-I were included to conduct hierarchical regression analysis. The main effect of psychopathy was significant after controlling gender (*b* = 0.31, *p* < 0.001). Psychopathy positively predicted proactive aggression. Also, the main effect of CFC-F (*b* = -0.24, *p* < 0.001) was significant after controlling gender and CFC-I but CFC-I (*b* = 0.025, *p* > 0.05) was not. CFC-F could negatively predicted proactive aggression but CFC-I could not. Hypothesis 1 was partly confirmed.

CFC-F and CFC-I also moderated the relationship between psychopathy and proactive aggression after controlling for the independent and interactive roles of gender. CFC-F negatively moderated the relation between psychopathy and proactive aggression, while CFC-I positively moderated it; this finding was consistent with Hypothesis 2. The hierarchical regression analysis results are presented in Table 2.

**Table 2:** Hierarchical regression models of predicting proactive aggression.

	Model 1		Model 2		Model 3		Model 4	
	*b*	*t*	*b*	*t*	*b*	*t*	*b*	*t*
**Gender**	0.18	5.82***	0.12	3.93***	0.10	3.45**	0.10	3.59***
**DT-P**			0.31	10.36***	0.28	9.66***	0.15	3.62***
**CFC-F**					-0.20	-6.89***	-0.20	-7.14***
**CFC-I**					0.056	1.96	0.073	2.60**
DT-P* Gender							0.19	4.60***
DT-P*CFC-F							-0.088	-3.11**
DT-P*CFC-I							0.089	3.14**
***ΔR***^***2***^	0.030		0.12		0.16		0.19	

DT-P, psychopathy; CFC-F, CFC-Future; CFC-I, CFC-Immediate;

*p < 0.05,

**p < 0.01,

*** *p* < 0.001.

**Fig 1:**
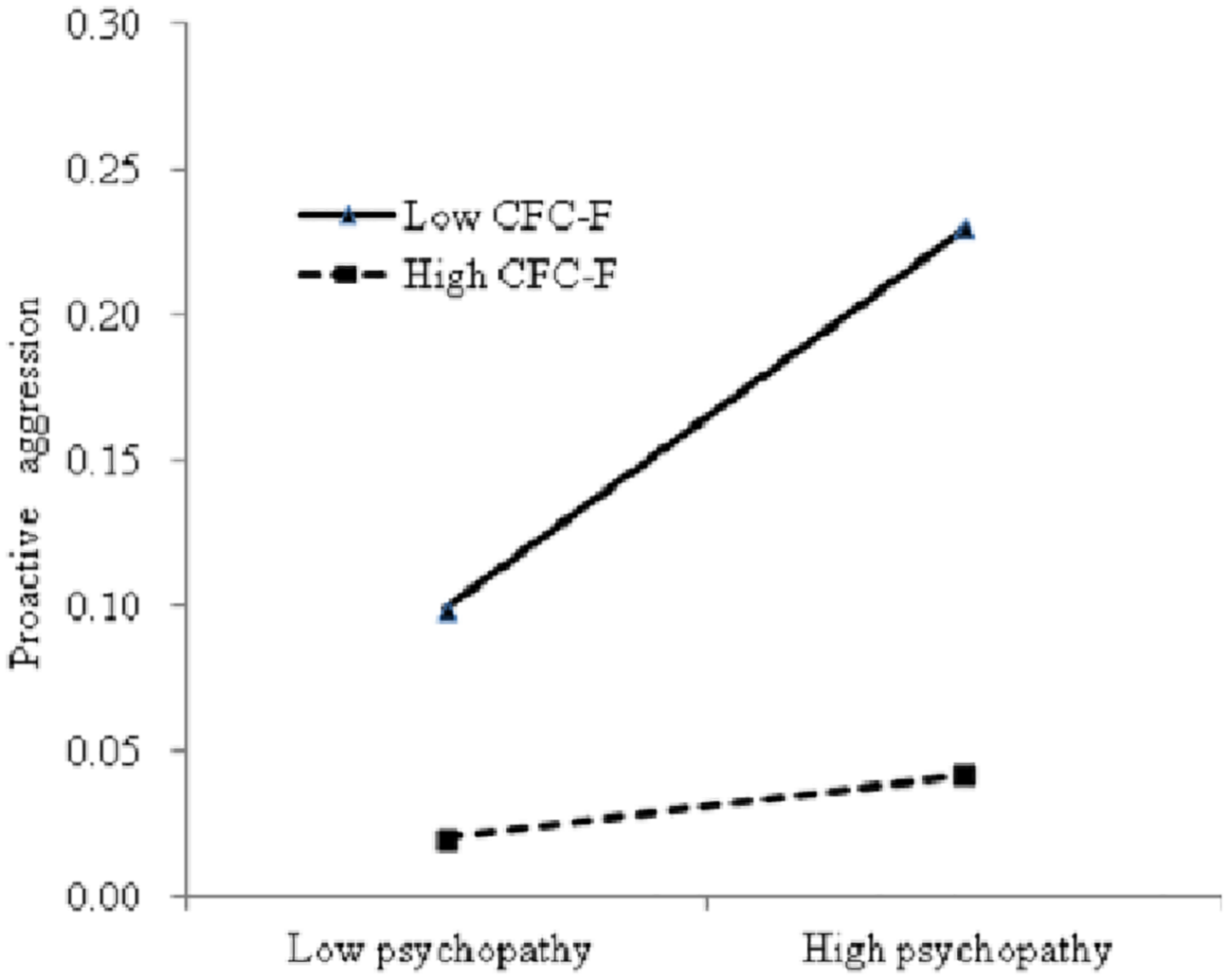
Interaction between CFC-F and psychopathy on proactive aggression. Note: CFC-F refers to CFC-Future.

According to the CFC-F scores, the participants were divided into three groups, and the moderating roles of the high (at +1 *SD* above the mean) and low (at –1 *SD* below the mean) CFC-F groups in the relation between psychopathy and proactive aggression were analyzed. The results in Fig 1 show that the predictive role of psychopathy on proactive aggression in the high CFC-F group (*b* = 0.17, *p* < 0.05) was lower than that in the low CFC-F group (*b* = 0.23, *p* = 0.006).

**Fig 2:**
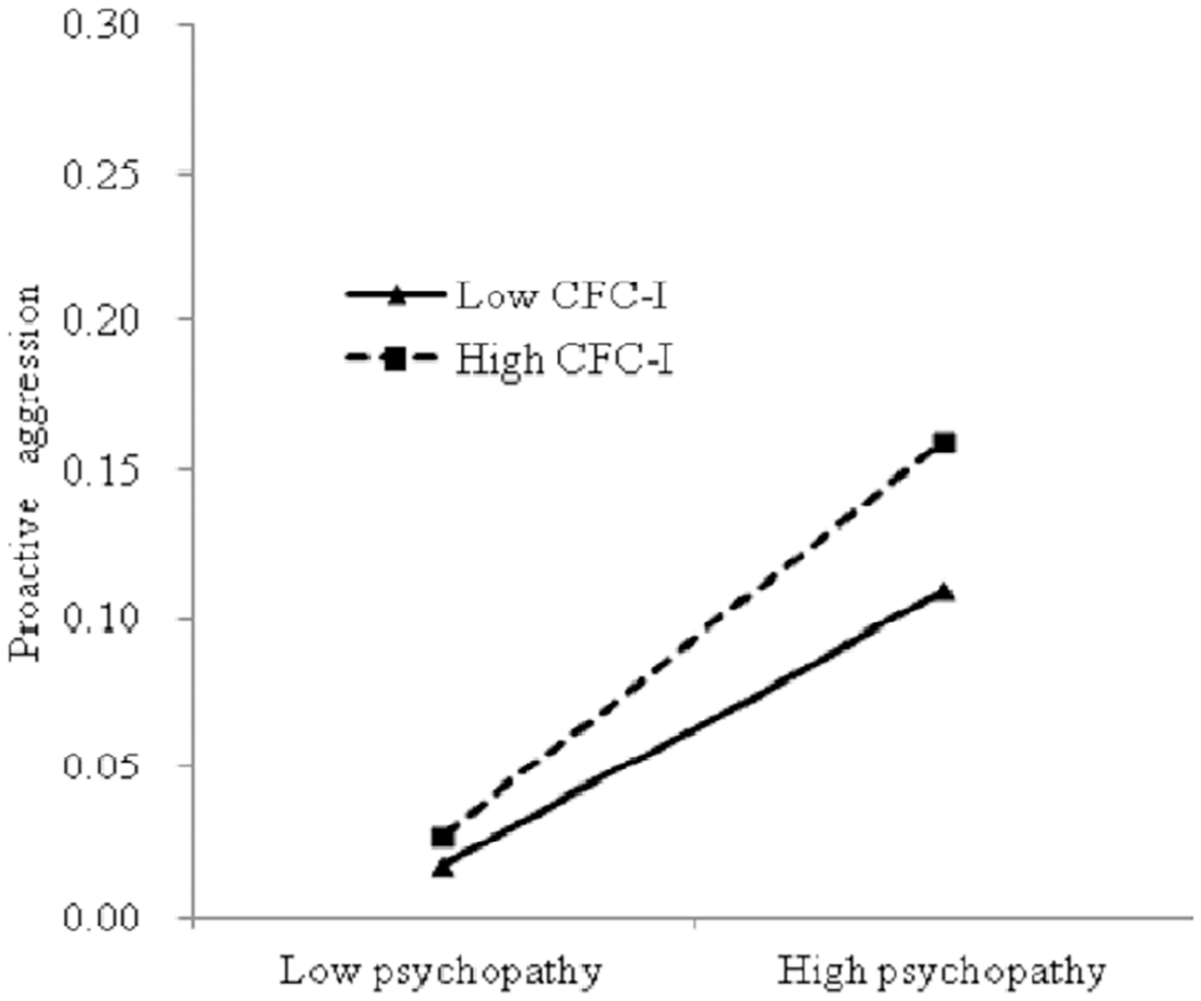
Interaction between CFC-I and psychopathy on proactive aggression. Note: CFC-I refers to CFC-Immediate.

Similar to the CFC-F analysis, the participants were divided into three groups according to their CFC-I scores. The results in Fig 2 show that the predictive role of psychopathy in proactive aggression in the low CFC-I group (*b* = 0.29, *p* < 0.001) was lower than that in the high CFC-I group (*b* = 0.33, *p* < 0.001).

In addition, the gender of the participants was significantly related to psychopathy, CFC-F and proactive aggression. Independent sample t-test analyses indicated that there were significant gender differences in psychopathy, *t* (1053) = -6.52, *p* < 0.001; CFC-F, *t* (1053) = 3.13, *p* = .002; and proactive aggression, *t* (1054) = -5.79, *p* < 0.001. The males scored higher on the psychopathy and proactive aggression scales than did the females, whereas the females scored higher on the CFC-F scales than did the males (Table 3). There was no significant age difference in any of the variables.

**Table 3:** Gender differences in the variables.

	Psychopathy		CFC-Future		Proactive aggression	
	*M*	*SD*	*M*	*SD*	*M*	*SD*
**Male**	2.40	1.08	3.46	0.59	0.14	0.26
**Female**	2.00	0.90	3.57	0.58	0.06	0.17

Our hypotheses were partially supported. Therefore, to ascertain the stability of the results in Study 1, we employed a cyber-aggression index to replicate the present findings. The research related to cyber-aggression has mostly focused on adolescents [18, 48] rather than adults and has ignored aggressive online comment behavior, a new cyber-aggressive behavior that may be used by adults online who comment on news websites [49]. Therefore, Study 2 aimed to examine the independent and moderating effects of the consideration of future consequences (CFC-I vs. CFC-F) on the relations between psychopathy and aggressive online comment behavior using an adult sample.

Although the research on the relationship between psychopathy and aggressive online comment behavior is limited, we may infer that psychopathy positively predicts aggressive online comment behavior. First, psychopathy predicts cyber-aggression/bullying [16–18]. Second, aggressive online comment behavior is one type of cyber-aggressive behavior.

## Study 2

The hypotheses of Study 2 were the same as those of Study 1.

### Method

#### Participants and procedure

We posted the questionnaires on the Sojump website, which is a professional online questionnaire survey, assessment and voting platform that helped us collect the data from adult participants who voluntarily completed the online surveys; we compensated participants a total of approximately 3000 RMB for these data. Three hundred fifty adult participants (162 males and 188 females) completed the survey. The participant ages ranged from 20 to 55 years (*M* = 30.38, *SD* = 6.34). The reported professions included students (12%, n = 42), public institution staff (14.9%, n = 52), community workers (2.6%, n = 9), business staff (66.9%, n = 234), individual households (2.9%, n = 10), and other employment (0.9%, n = 3). The educational backgrounds included high school (1.7%, n = 6), junior college (12.9%, n = 45), bachelor’s degree (76%, n = 266) and graduate degree (9.4%, n = 33).

The participants voluntarily completed the online surveys, including the psychopathy subscale of the Dirty Dozen scale, the CFC scale, and the aggressive online comment behavior scale. We used procedural remedies to control likely common method biases, which was the same approach as that used in Study 1. Finally, the participants provided their demographic data. This study was approved by the Ethics Committee at the Faculty of Psychology, Southwest University, China.

#### Materials

The psychopathy subscale was the same as that used in Study 1. The Cronbach’s *α* for the present sample was 0.84.

The CFC scale was the same as that used in Study 1. The Cronbach’s *α* of the two subscales for the present sample was 0.79 for the CFC-I and 0.64 for the CFC-F.

Aggressive online comment behavior scale. Based on the theoretical framework of electronic aggression by Pyżalski [48], a four-item aggressive online comment behavior scale was created, and it included online aggression against random victims, against vulnerable individuals (e.g., rural migrant workers in cities), against groups (e.g., an ethnic or religious group), and against celebrities (e.g., singers or sports stars). The participants were instructed to report the frequency of the aggressive comments they had made on news websites in the past three months, 1 = never, 2 = once, 3 = 2–4 times, 4 = 5–7 times, 5 = 8–10 times, and 6 = more than 10 times. All four items were Varimax-rotated and classified into one dimension, which accounted for 68.54% of the accumulation contribution rate. The Cronbach’s α of the scale for the present sample was 0.85.

Demographic questionnaire. The questionnaire included four items, namely, gender, age, education, and employment.

#### Statistical analysis

We used statistical remedies, which was the same approach as that used in Study 1. The results indicated four factors with eigenvalues greater than 1.0 and the first factor accounted for 18.71% of the accumulation contribution rate (< 40%). Our results indicate that common method biases are not a problem in Study 2. The statistical analysis was the same as that used in Study 1, with factorial analysis also conducted for the aggressive online comment behavior scale.

### Results and discussion

Descriptive analyses were performed, and a correlation matrix was prepared to assess the overall patterns of psychopathy, CFC-I, CFC-F, and aggressive online comment behavior. Correlation coefficients, the means, and standard deviations are presented in Table 4.

**Table 4:** Zero-order correlations, means, and standard deviations.

	**1**	**2**	**3**	**4**	**5**	**6**
**1 DT-P**	1					
**2 CFC-I**	0.31**	1				
**3 CFC-F**	-0.23**	-0.18**	1			
**4 AGGRE**	0.55**	0.24**	-0.16**	1		
**5 Gender**	-0.16*	-0.11*	0.14*	-0.08	1	
**6 Age**	-0.14**	-0.02	0.13*	-0.08	-0.12*	1
***M***	2.33	2.92	3.71	1.87	1.54	30.38
***SD***	1.22	0.77	0.57	0.94	0.50	6.34

DT-P, psychopathy; CFC-F, CFC-Future; CFC-I, CFC-Immediate; AGGRE, aggressive online comment behavior;

*p < 0.05,

**p < 0.01.

Table 4 showed that psychopathy and CFC-I were positively related but CFC-F negatively related to aggressive online comment behavior. To test the main and moderating effects, gender, psychopathy, CFC-F and CFC-I were included to conduct independently and hierarchical regression analysis. The main effects of psychopathy were significant after controlling gender (*b* = 0.56, *p* < 0.001). Psychopathy positively predicted aggressive online comment behaviors. Also, the main effect of CFC-F (*b* = -0.12, *p* < 0.05) was significant after controlling gender and CFC-I. the main effect of CFC-I (*b* = 0.22, *p*< 0.001) was significant after controlling gender and CFC-F. CFC-F can negatively but CFC-I positively predicted aggressive online comment behaviors. Hypothesis 1 was confirmed.

CFC-F and CFC-I also moderated the relationship between psychopathy and aggressive online comment behaviors after controlling for the independent and interactive roles of gender. CFC-F negatively moderated the relationship between psychopathy and aggressive online comment behaviors, but CFC-I positively moderated it; this finding was consistent with Hypothesis 2. The hierarchical regression analysis results are presented in Table 5.

**Table 5:** Hierarchical regression models predicting aggressive online comment behaviors.

	Model 1		Model 2		Model 3		Model 4	
	*b*	*t*	*b*	*t*	*b*	*t*	*b*	*t*
**Gender**	-0.080	-1.50	0.008	0.17	0.015	0.34	0.005	0.12
**DT-P**			0.56	12.26***	0.53	10.94***	0.31	2.05*
**CFC-F**					-0.027	-0.57	-0.031	-0.69
**CFC-I**					0.077	1.62	0.12	2.60*
DT-P* Gender							0.11	0.79
DT-P*CFC-F							-0.13	-2.96**
DT-P*CFC-I							0.19	3.86***
***ΔR***^***2***^	0.004		0.30		0.31		0.34	

DT-P, psychopathy; CFC-F, CFC-Future; CFC-I, CFC-Immediate;

*p < 0.05,

**p < 0.01,

*** *p* < 0.001.

**Fig 3:**
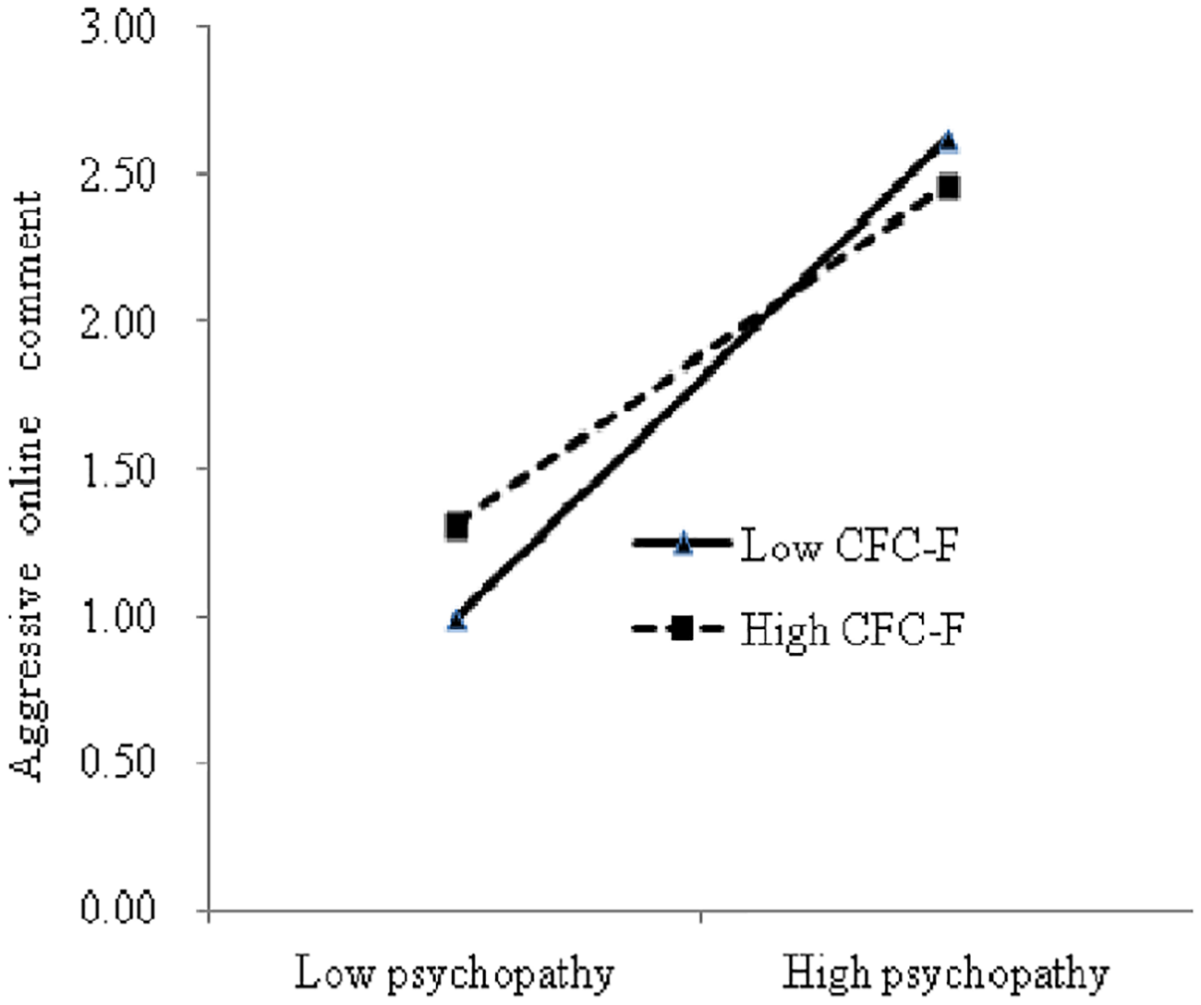
Interaction between CFC-F and psychopathy on aggressive online comment behavior. Note: CFC-F refers to CFC-Future.

The participants were divided into three groups according to CFC-F scores, and the moderating roles of the high (at +1 *SD* above the mean) and low (at –1 *SD* below the mean) CFC-F groups between psychopathy and aggressive online comment behavior were analyzed. The results in Fig 3 show that the predictive role of psychopathy on aggressive online comment behavior in the high CFC-F group (*b* = 0.62, *p* < 0.001) was lower than that in the low CFC-F group (*b* = 0.74, *p* < 0.001).

**Fig 4:**
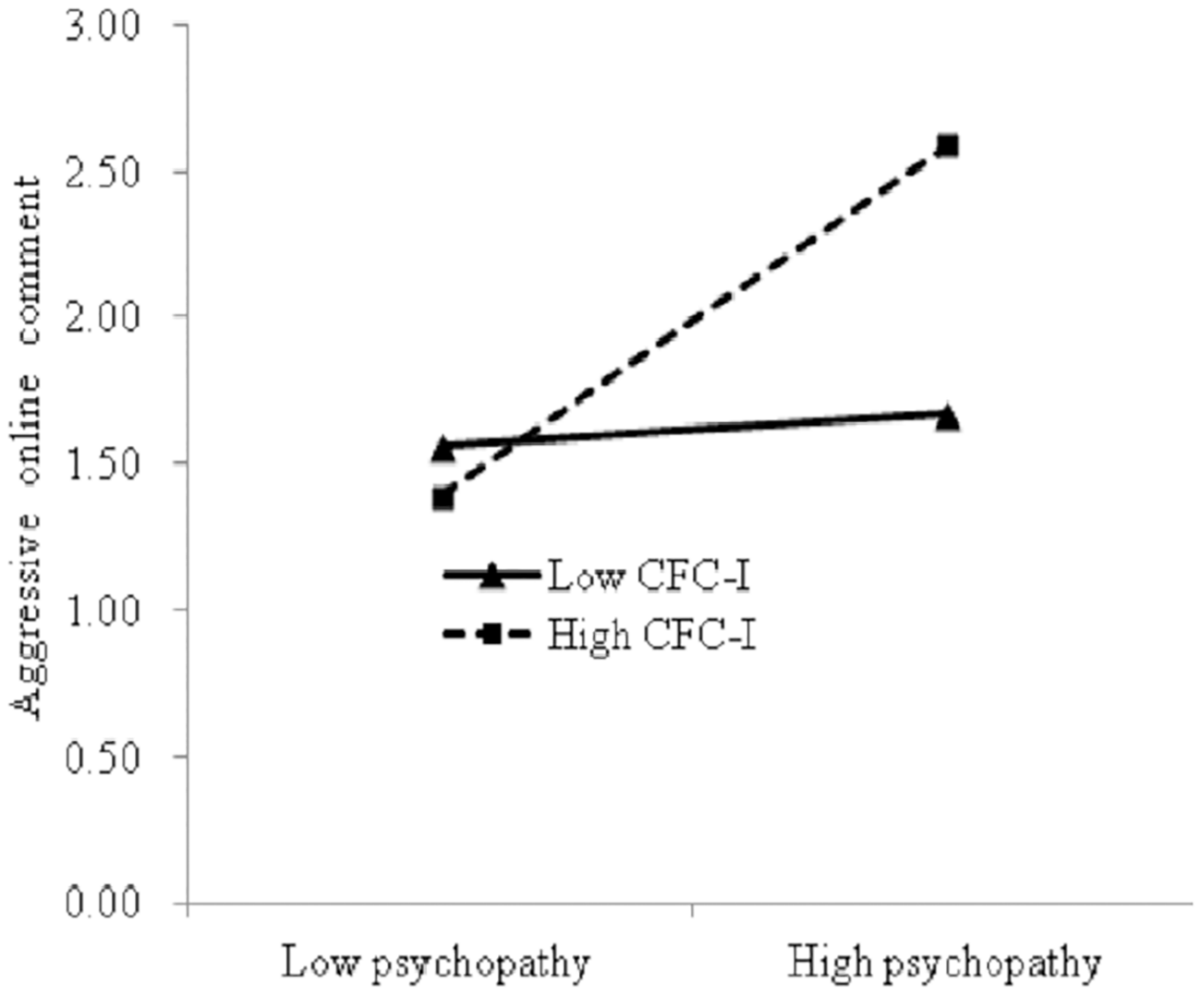
Interaction between CFC-I and psychopathy on aggressive online comment behavior. Note: CFC-I refers to CFC-Immediate.

Similar to the CFC-F analysis, the participants were divided into three groups according to their CFC-I scores. Fig 4 shows that psychopathy significantly predicted aggressive online comment behavior in the high CFC-I group (*b* = 0.61, *p* < 0.001), whereas psychopathy did not predict aggressive online comment behavior in the low CFC-I group (*b* = 0.050, *p* > 0.05).

The findings of Study 2 mostly confirmed those of Study 1 regarding the independent and interactive effects of psychopathy and the consideration of future consequences (CFC-I vs. CFC-F) on aggressive behaviors.

In addition, participant age was positively related to psychopathy and CFC-F. With an increase in age, the participant scores on psychopathy decreased, whereas the participant scores on the CFC-F increased. Furthermore, participant gender was positively related to psychopathy, CFC-I and CFC-F. Independent sample t-test analyses indicated that there were significant gender differences in psychopathy, *t* (348) = 2.99, *p* < 0.01; CFC-I, *t* (348) = 2.17, *p* < 0.05; and CFC-F, *t* (348) = -2.58, *p* = 0.01. The males scored higher on the psychopathy and CFC-I scales than did the females, whereas the females scored higher on the CFC-F scales (Table 6). There were no significant gender or age differences in aggressive online comment behavior.

**Table 6:** Gender differences among the variables.

	Psychopathy		CFC-Future		CFC-Immediate	
	*M*	*SD*	*M*	*SD*	*M*	*SD*
**Male**	2.54	1.25	3.63	0.58	3.02	0.69
**Female**	2.15	1.17	3.78	0.56	2.84	0.83

## General discussion

The present findings replicated previous results, in which psychopathy significantly predicted aggression [5–7]. The results also confirmed that CFC played a protective role against aggression. However, most importantly, the current study extended previous research by examining CFC, which was expected to influence the relationship between psychopathy and aggression.

### Primary findings

The results supported that psychopathy was a robust predictor of aggressive behavior [5, 6]. Psychopathy was positively related to proactive aggression (Study 1), which was consistent with the findings of existing research [1, 14, 50]. Psychopathy also positively predicted aggressive online comment behavior (Study 2), which was consistent with the findings of related cyber-aggression/bullying research [15–18]. Individuals with high psychopathy scores were prone to implementing more proactive aggression (Study 1) and expected to leave more aggressive online comments on news websites (Study 2).

CFC-F negatively predicted proactive aggression (Study 1) and aggressive online comment behavior (Study 2); this finding was consistent with the results of related studies [30, 32–35]. CFC-I positively predicted aggressive online comment behavior (Study 2); this finding was consistent with the results of related studies [31, 35, 51]. The findings indicate that if an individual considers the future consequences or look-downs on the immediate consequences of one’s actions, the individual may react prudently, avoiding excessive aggression despite high scores in psychopathy. However, CFC-I did not predict proactive aggression (Study 1). Therefore, future research must examine the different relations between CFC-I and different kinds of aggression.

Across the two studies, CFC-F and CFC-I moderated the relationship between psychopathy and aggressive behavior. Individuals who had high psychopathy scores with simultaneously high CFC-F or low CFC-I scores were less likely to behave aggressively. Therefore, high CFC-F levels and low CFC-I levels buffered the predictive power of psychopathy on aggressive behavior. The results were consistent with those of related research [43, 52] and suggest a potential protective role of high CFC-F or low CFC-I against the influence of psychopathy on aggression.

### Implications and contributions

This study has several implications for existing studies. First, substantial research has focused on why and how psychopathy (as a personality trait) predicts all types of aggression; however, few studies have explored how to buffer the linkage between psychopathy and aggression [41]. This study examined the buffering effects of CFC in the linkage between psychopathy and aggression. This finding is the first unique contribution of the present study. Second, a new aggressive index (aggressive online comment behavior) was employed in Study 2. Thus, this study extended previous studies with respect to psychopathy and aggressive behavior. In addition, CFC-F and CFC-I both moderated the relationship between psychopathy and aggression; however, the moderating effects were in different directions. The findings suggested that research on the bi-dimensional construct of CFC was valuable.

More importantly, this study provides several implications for the prevention and intervention of aggressive behavior. The finding that CFC-F and CFC-I moderate the impact of psychopathy on aggression indicates that psychopathy is not invariably linked to increased aggression. Thus, we can cultivate positive personality traits (i.e., CFC) against aggression related to psychopathy. Moreover, as the consideration for future consequences mirrors future orientation, future orientation may be shaped through intervention [53, 54]. Interventions aimed at attenuating aggression can focus on convincing individuals to care about long-term consequences and simultaneously decrease individuals’ concerns with the immediate consequences of aggression. Thus, training in consequence cognition may be conducted to help individuals understand the influence of the immediate and distant consequences of aggression on themselves and other individuals, therefore reducing aggressive behavior.

### Limitations and future research directions

Several limitations should be considered when interpreting the results. First, we just evaluated aggression and other variables via self-report method. Future research should be strengthened by the inclusion of other techniques, such as the Taylor Aggression Paradigm [55], to measure actual aggressive behavior. We could also employ multiple informants (such as peer or colleagues) and multiple research design (such as experimental or longitudinal design) to decrease likely common method biases. Second, the moderation effects of CFC-F and CFC-I were small in Study 1, although the effects were significant. Therefore, additional evidence for these results should be obtained in the future. Third, the participants in Study 2 voluntarily participated in this study, and the author did not control their demographic data. Therefore, the sample mainly comprised business staff (66.9%) and cannot represent all adults who comment on news websites. Additional similar studies should be conducted to ascertain the stability of the results. Fourth, we used the briefest measure of the psychopathy subscale of the Dirty Dozen scale [45], as opposed to the multiple dimensions psychopathy scale [56], which may result in a loss of information from the meaningful facets of these scales. Future research should employ these scales to examine which dimensions of psychopathy may be influenced by the moderating role of CFC. Finally, CFC is a personality trait and is closely related to but different from consequence cognitions. Consequences may involve several aspects, such as the consequences of victims, perpetrators, groups, and even society. Different consequence cognitions may affect the moderating role between psychopathic traits and aggressive behaviors. For example, the predictive role of aggressive behavior may be different between a cognition on the negative consequence to offenders and a cognition on the negative consequence to victims, and vice versa. This issue deserves attention in future research.

## Conclusion

It is important for researchers to determine which personality traits predict aggression; however, it is equally important to examine which individual variables may buffer or break the connections between “dark” personalities and aggression. This article explored the protective roles of CFC (CFC-Future and CFC-Immediate) in attenuating aggression triggered by psychopathy using proactive aggression and cyber-aggression behavior indexes. The results across the two studies demonstrated that psychopathy and CFC-Future significantly predict aggressive behavior, and the relationship between psychopathy and aggressive behaviors was moderated by CFC-Future and CFC-Immediate. Individuals with high psychopathy scores and simultaneously high CFC-Future scores or low CFC-Immediate scores have less proactive aggression and fewer aggressive online comment behaviors. Thus, CFC serves as a buffer against aggression triggered by psychopathy. Training for CFC in aggressive behavior may be a successful method to buffer or decrease aggressive behavior triggered by “dark” psychopathic traits.
